# Penalized partial least squares for pleiotropy

**DOI:** 10.1186/s12859-021-03968-1

**Published:** 2021-02-24

**Authors:** Camilo Broc, Therese Truong, Benoit Liquet

**Affiliations:** 1 LIST, CEA, Laboratory for Data Sciences and Decision (Digiteo), Gif-sur-Yvette, France; 2 CNRS, Laboratoire de Mathématiques et de leurs Applications de PAU E2S UPPA, Pau, France; 3 UVSQ, Inserm, CESP, Université Paris-Saclay, 94807 Villejuif, France; 4 Institut Gustave Roussy, 94805 Villejuif, France; 5 Department of Mathematics and Statistics, Macquarie University, Sydney, Australia

**Keywords:** Genetic epidemiology, High dimensional data, Lasso Penalization, Meta-analysis, Oncology, Partial Least Square, Pathway analysis, Pleiotropy, Sparse methods, Variable selection

## Abstract

**Background:**

The increasing number of genome-wide association studies (GWAS) has revealed several loci that are associated to multiple distinct phenotypes, suggesting the existence of pleiotropic effects. Highlighting these cross-phenotype genetic associations could help to identify and understand common biological mechanisms underlying some diseases. Common approaches test the association between genetic variants and multiple traits at the SNP level. In this paper, we propose a novel gene- and a pathway-level approach in the case where several independent GWAS on independent traits are available. The method is based on a generalization of the sparse group Partial Least Squares (sgPLS) to take into account groups of variables, and a Lasso penalization that links all independent data sets. This method, called joint-sgPLS, is able to convincingly detect signal at the variable level and at the group level.

**Results:**

Our method has the advantage to propose a global readable model while coping with the architecture of data. It can outperform traditional methods and provides a wider insight in terms of a priori information. We compared the performance of the proposed method to other benchmark methods on simulated data and gave an example of application on real data with the aim to highlight common susceptibility variants to breast and thyroid cancers.

**Conclusion:**

The joint-sgPLS shows interesting properties for detecting a signal. As an extension of the PLS, the method is suited for data with a large number of variables. The choice of Lasso penalization copes with architectures of groups of variables and observations sets. Furthermore, although the method has been applied to a genetic study, its formulation is adapted to any data with high number of variables and an exposed a priori architecture in other application fields.

## Background

Genome-wide association studies (GWAS) have identified numerous genetic markers linked to multiple phenotypes, suggesting the existence of pleiotropy that occurs when a single variant or gene can influence several phenotype traits [1–4]. Highlighting pleiotropy provides opportunities for understanding the shared genetic underpinnings among associated diseases. However genetic information may be spread among different studies (a) because the signal is small and larger sample sizes can increase the ability of detection (b) because in the case of rare phenotype, analyses require to study distinct data sets corresponding to different phenotypes. Therefore combining data across studies is necessary for cross-phenotype or pleiotropic association analyses. Combining data across studies on different phenotypes could also permit to increase statistical power to detect new signals weakly associated to several phenotypes. This leads to consider data sets from different sources, having common genotype data, but which phenotype traits may differ from one study to another.

In this article, we are interested in meta-analysis methods dealing with data from independent studies. Genetic information comes from single nucleotide polymorphisms (SNP). Genes are defined by a set of SNPs grouped in the same location in the genetic sequence. Pathways are groups of genes involved in a common biological mechanism. Genetic analyses aim at testing the association between genetic variants and phenotypes at the SNP-, gene- or pathway-level. Hence, information about independent data sets gives an architecture in terms of observation sets while information about either genes or pathways gives an architecture in term of groups of variables. The challenge of pleiotropy is then to take advantage of these architectures.

In addition, possible biases between observation sets can be induced in genetic studies especially due to differences of studied population, used technologies or experimented protocols. Those called “batch effects” are a common problem for meta-analyses [5], and methods for pleiotropy must take it into account. Furthermore, such methods must cope with the case where a genetic variable have a positive effect on one trait and a negative effect on another traits. Those opposite effects cannot be highlighted by standard meta-analysis methods [6, 7].

Various statistical methods were proposed for gene set analysis or to analyze pleiotropy. Recent pleiotropy analyses rely on statistical methods coming from gene set analysis combined with a meta-analysis [1, 8–10]. A non-exhaustive list of gene set methods can be given. Burden test and variance component tests have been developed to analyse rare variants [7, 11–13]. Alternatively, dimensionality reduction methods [14, 15] and Bayesian models have also been largely exploited [16, 17]. We can also cite pairwise similarity based model [18], U-statistic models [19, 20], linear model family methods [21, 22] and network-based methods [23]. Furthermore, other omics fields are rising [24–27] and methods for genomics are often reused in those analyses [28].

We aim at integrating the meta-analysis perspective in cases of distinct data set to a gene set method framework. An extension of the sparse Partial Least Square (sPLS) method suited for meta-analysis for pleiotropy is proposed. It deals with observation sets and group of variables information while taking into account the possibility of opposite effects, i.e cases where a genetic variable has a positive effect on one trait and negative effect on other trait. As a sPLS family method, it can cope with the high number of variables. The method formulates at the same time a group-lasso resp. a joint-lasso penalization to represent the group of variables resp. the sets of observations.

PLS is a dimensionality reduction method developed by Wold [29] and that has been widely used for the analysis of data with large number of variables [30]. Applications have been done outside of genetic studies, for instance in chemometry [31] or for neuroimaging [32]. Unlike, its cousin method (PCA), the Principal Component Analysis (PCA) [33], the PLS deals with two blocks of data and this is used for genotype-phenotype analyses. Moreover its sparse extension using Lasso penalization has been successful at providing readable models [34]. Especially sparse group Partial Least Square can take into account group of variables as a priori information [35, 36]. For different group of studies an alternative Lasso penalization has been proposed by Obozinski [37] for a linear regression to deal with data made of different sets of observations. An adaptation of the Lasso penalization, the joint-sgPLS, has recently been proposed for the PLS [38], answering the specific of both groups of variables and sets of observations. In this article, we exploit the same idea to leverage pleiotropy effects, especially because the method copes with the challenge of detecting small possible opposite effects.

The method is compared to two well established statistical methods in genetic studies. The first one, ASSET [6] extends standard fixed-effects meta-analysis methods for detecting effects in opposite directions from a same genetic trait. The second one metaSKAT [7] permits to carry out gene-based meta-analysis extends SKAT and SKAT-o methods for meta-analyses.

The developed statistical approaches will be applied to real dataset for enriching our insights about the genetic mechanisms of thyroid and breast cancer types. We are interested into exploring gene-level and pathway-level associations for each cancer type as well as for both cancer types together.

## Methods

### Notations

**Fig. 1:**
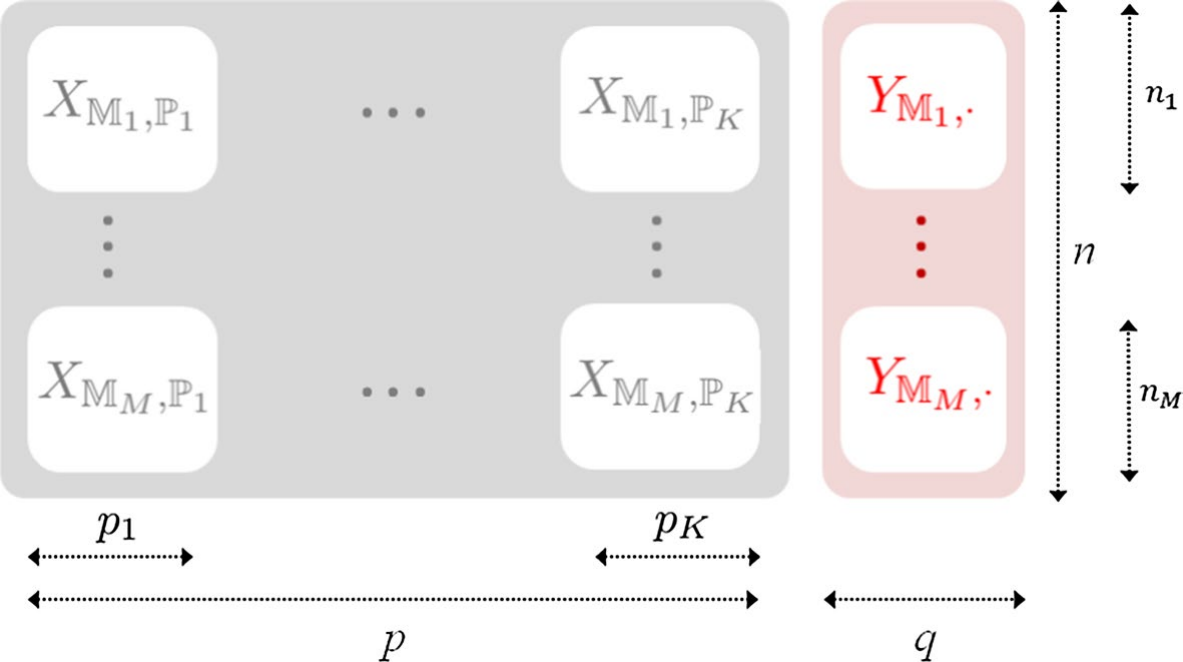
Illustration of data structured by groups of variables and sets of observations. Variables and observations are assumed to be ordered by resp. groups of variables and observations sets. The notation *p* represents the number of variables of matrix *X*, *q* the number of variables of matrix *Y*, *n* is the number of observations. $n_1, \cdots , n_M$ are the resp. number of observations of each observation set. $p_1, \cdots , p_K$ are the resp. number of variables in each group of variables

Data are represented by $X \in \mathbb {R}^{n \times p}$ and $Y \in \mathbb {R}^{n \times q}$, two matrices, representing *n* observations of *p* predictors and *q* independent variables. The Frobenius norm on matrices is denoted $\left\| ~ ~ \right\| _F$. We note $X^T$ the transpose matrix of *X* and the cardinal of a set *S* is noted $\# S$. The positive value of a real number *x* is noted $(x)_+ = \frac{|x|+x}{2}$ and is equal to the number if the number is positive and equal to zero otherwise. In general, observation sets can represent the fact that different sets of observations come from different sources and must be analyzed accordingly. For instance, data coming from different studies may present biases. Variables groups can represent a set of variables that are known or suspected to be part of a same signal. For instance, in genetics a gene defines an established group of SNP variables and pathways define established group of genes. Let us consider *M* different sets of observations in the data. Noting, for $m \in \mathbb {N}$, $\mathbb {M}_m$ a subset of $\{1, \dots , n\}$, let $\mathbb {M}=(\mathbb {M}_m)_{m=1..M}$ be a partition of $\{ 1,...,n \}$ corresponding to the observation sets. We note $\# \mathbb {M}_m=n_m$. Row blocks are defined by this partition. Let us consider that variables are gathered in *K* groups. Let $\mathbb {P}=(\mathbb {P}_k)_{k=1..K}$ be a partition of $\{ 1,...,p \}$ corresponding to this variable group architecture. We note $\# \mathbb {P}_k=p_k$. We then we have $\sum _{k=1}^K p_k =p$. Column blocks are defined by this partitions. Both observation set architecture and variable group architecture can be defined at the same time as shown in Fig. 1. For matrices, the notation $\cdot$ is used to refer to blocks of matrices. For instance $X_{\cdot , \mathbb {P}_k}$ is the block of matrix of *X* corresponding the columns of the *k*-th group of variables and $X_{ \mathbb {M}_m,\cdot }$ is the block of matrix of *X* corresponding the columns of the *m*-th set of observations.

### Sparse Partial Least Square for structured data

In the literature, several formulations of the PLS exist [39]. While they can have similar performances [40], PLS1 [41] has prevailed in last developments [35, 40, 41]. In the scope of this article, this formulation has been chosen in order to be able to pursue the path of previous methods. PLS finds successively couples of vector $\{u_1,v_1 \} , \dots , \{u_r,v_r \}$ for $r < \text{ rank }(X)$, where the couples are composed of vectors of length resp. *p* and *q*, maximizing $Cov(Xu_i,Yv_i) \text{ for } \text{ any } i \in \{1,\dots ,r\}$, under the constraint that $u_1, \dots , u_r$ are related to orthogonal families of components [29]. It can be solved considering successive maximization problems [42], for $h \in \{1, \dots , r\}$

1 $$\begin{aligned} \underset{||u_h||_2=||v_h||_2=1}{ \text{ max }} Cov(X^{(h-1)} u_h,Y^{(h-1)} v_h), \end{aligned}$$

where $X_0 =X$, $Y_0=Y$ and $X^{(h-1)}$, $Y^{(h-1)}$ are deflated matrices computed from $u^{(h-1)}$,$v^{(h-1)}$, $X^{(h-2)}$, $Y^{(h-2)}$ for $h \in \{2, \dots , r \}$. The deflation depends on the PLS mode that is chosen [29, 43]. In the following, the notation *h* is removed in order to simplify the formulation because we are interested in only one of the *r* steps of the PLS.

The sparse PLS (sPLS) propose to add a penalization to the loading vectors *u* and *v*. The following equivalence is used:

2 $$\begin{aligned} \underset{||u||_2=||v||_2=1, u \in \mathbb {R}^p, v \in \mathbb {R}^q }{ \text{ argmax }} Cov(Xu,Yv) = \underset{||u||_2=||v||_2=1, u \in \mathbb {R}^p, v \in \mathbb {R}^q }{ \text{ argmin }} \left\| X^TY - uv^T \right\| _F^2 \end{aligned}$$

and the proof can be found in [35].

The sPLS [42] can be written as

3 $$\begin{aligned} \{ u^{(opt)}, v^{(opt)} \} = \underset{||u||_2=||v||_2=1, u \in \mathbb {R}^p, v \in \mathbb {R}^q}{\text{ argmin }} \left\| X^T Y- u v^T \right\| _{F}^2 + \underbrace{\lambda P(u)}_{ \underset{ \text{ for } \text{ sparse } \text{ PLS } }{\text{ Lasso } \text{ Penalty } \text{ term }} } . \end{aligned}$$

The sparse PLS introduces a penalization in this formulation of the problem. The penalty $P( \cdot )$ forces smallest participation to *u* to be set to zero. The parameter controlling the degree of sparsity in the model is $\lambda$. In the presented formula the sparsity is applied only to the vector *u*, but a similar penalization can be defined for *v*. In the context of this article we treat only the penalization of *u* but all the results stand also for a *v* penalization.

#### *Remark 1*

Before analysis, the X and Y matrices are transformed by subtracting their column averages. Scaling each column by their mean and standard deviation is also often recommended [44]. Thus, the cross-product matrix $X^T Y$ is proportional to the empirical covariance between X- and Y-variables when the columns of X and Y are centered. When the columns are standardized, $X^T Y$ is proportional to the empirical correlations between X- and Y-variables. In this article the standardization is an important step to overcome the issue of the “batch effect” or to aggregate observations from different studies. The point has been discussed in [38].

#### *Remark 2*

Presented framework deals with the estimation of a pair of weight vectors (*u*, *v*), which is the main contribution of the method in terms of methodology. This estimation step can then be included in the global framework of PLS with the deflation steps for modeling several components.

### Extensions of the sparse Partial Least Square

In the following, extensions of the sPLS taking into account an observation or/and variable set architectures are presented. The last method has been recently developed [38] and deals with both kinds of architecture. It is the main topic of the article. Proposed model is an extension of the multigroup sPLS proposed by Eslami et al. [40].

In order to cope with the architectures, sgPLS has been proposed [35]:

4 $$\begin{aligned} \begin{aligned} \{ u^{(opt)}, v^{(opt)} \} &= \underset{||u||_2=||v||_2=1, u \in \mathbb {R}^p, v \in \mathbb {R}^q}{\text{ argmin }} \left\| Z- u v^T \right\| _{F}^2 + \lambda \left( 1- \alpha \right) P_{group}(u)+ \\ \lambda \alpha P_{variable}(u) \\ & \quad \text{ with } P_{group}(u) =\sum _{k=1}^{K} \sqrt{p_k} \left\| u_{\mathbb {P}_k} \right\| _2 \text{, } P_{variable}(u) =\sum _{i=1}^{p} \left\| u_{i} \right\| _2 \\ \text{ and } Z = X^T Y. \end{aligned} \end{aligned}$$

where the loading vectors *u* and *v* are composed of resp. *p* and *q* elements. Penalization $P_{variable}$ shrinks variables individually towards zero whereas penalization $P_{group}$ shrinks whole groups of variables towards zero. The parameter driving the degree of sparsity of the model is $\lambda$ whereas the parameter controlling the balance between both kinds of sparsity is $\alpha$. In this model elements of *u* corresponding to least relevant variables and least relevant groups of variables are set to zero.

An extension using the joint Lasso penalization from Obozinski ( [37]) has been proposed [38]. This method is the object of study of this article. Its formulation for the sgPLS is:

5 $$\begin{aligned} \begin{aligned} &\{ U^{(opt)}, V^{(opt)} \} = \underset{\underset{||U_{\cdot ,m}||_2=||V_{\cdot ,m}||_2 =1 \text{ for } m \in \{ 1, \cdots , M \}}{ U \in \mathbb {R}^{p \times M} \text{ and } V \in \mathbb {R}^{q \times M} } }{\text{ argmin }} \sum _{m=1}^{M} \left\| Z^{(m)} -U_{\cdot ,m} {V_{\cdot ,m}}^T \right\| _{F}^2 \\ &\quad +\,\lambda \left( 1- \alpha \right) P_{group}(U)+ \lambda \alpha P_{variable}(U) \\ &\text{ with } P_{group}(U) =\sum _{k=1}^{K} \sqrt{p_k} \left\| U_{\mathbb {P}_k,\cdot } \right\| _F \text{, } P_{variable}(U) =\sum _{i=1}^{p} \left\| U_{i,\cdot } \right\| _2 \\ & \text{ and } Z^{(m)}=X_{\mathbb {M}_m, \cdot }^{T} Y_{\mathbb {M}_m, \cdot }, \end{aligned} \end{aligned}$$

where the set of loadings *U* is composed of $p \times m$ elements (*p* elements per $U_{\cdot ,m}$). The set of loadings *V* is composed of $q \times m$ elements (*q* elements per $V_{\cdot ,m}$). In this model elements of *U* corresponding to least relevant variables and least relevant group of variables are set to zero. Variables and groups of variables corresponding to least participating variables are set to zero for all $U_{\cdot ,m}$, $m \in \{1,\dots ,M \}$ at the same time.

The solution of Eq. 5 is:

6a $$\begin{aligned} U^{(opt)}_{\mathbb {P}_k,\cdot } = U^{(1)}_{\mathbb {P}_k,\cdot } \left( 1- \frac{\lambda \left( 1- \alpha ) \right) }{2 \left\| U^{(1)}_{(\mathbb {P}_k,\cdot )} \right\| _F }\right) _+ \end{aligned}$$

6b $$\begin{aligned} \text{ with } U^{(1)}_{i,\cdot } = U^{(0)}_{i,\cdot } \left( 1- \frac{\lambda \alpha }{2 \left\| U^{(0)}_{i,\cdot } \right\| _2} \right) _+ \end{aligned}$$

6c $$\begin{aligned} \text{ and } \text{ with } U^{(0)}_{\cdot ,m} = X_{\mathbb {M},\cdot }^TY_{\mathbb {M},\cdot } \end{aligned}$$

where the positive value of a real number *x* is noted $(x)_+ = \frac{|x|+x}{2}$.

The solution is computed in 3 steps. First step (Eq. 6a) represents the solution of simple PLS for each M studies separately. Second step (Eq. 6b) applies sparsity on each variable for all studies at once. Third step (Eq. 6c) sets a sparsity on each group of variables for all studies at once. For all sparse methods, optimal parameters driving the penalization ($\lambda$ and $\alpha$) must be chosen. A K-fold cross-validation is used here. For each set of penalization parameters that must be tested:

- Observations are split into a partition of *L* samples: $\{\mathbb {S}_1 , \cdots , \mathbb {S}_{L}\}$. For a qualitative outcome, samples are chosen respecting the proportion of population of the outcome. For $l \in \{1 , \cdots , L \}$, the subset of $\{1, \cdots , n\}$ where $\mathbb {S}_{l}$ is omitted is noted $\mathbb {S}_{-l}$.
- For $l \in \{1, \cdots , L\}$, a model is performed on $X_{\mathbb {S}_{-l},\cdot }$ and $Y_{\mathbb {S}_{-l},\cdot }$. From this model a prediction is performed on $X_{\mathbb {S}_{l},\cdot }$ which gives a prediction ${\hat{Y}}_{\mathbb {S}_{-l},\cdot }$. Prediction error is computed comparing ${\hat{Y}}_{\mathbb {S}_{-l},\cdot }$ and ${Y}_{\mathbb {S}_{-l},\cdot }$. For qualitative outcome, a miss-classification rate is computed. For a quantitative outcome a L2-norm is computed. For multivariate outcome, the mean prediction over each variable outcome is computed.
- The mean of prediction errors over the *L* models is computed.

The set of parameters corresponding to the lowest error of prediction over the procedure above is selected. An example of the procedure can be found in the implementation of many extensions of the sPLS [35, 36, 40].

The K-fold procedure relies on the prediction performances. However, if the signal is too small, prediction can be poor and the calculation of optimal parameters can be problematic in a cross-validation framework. Other Lasso penalization methods have struggled when the number of variables is large [42, 45]. Due to the large number of variables in genomic data, the difference in term of prediction performance is not large enough to highlight one clear choice of penalization parameter. In this article, an alternative bootstrap strategy is proposed: sgPLS and joint-sgPLS are evaluated with given parameters on the data. Then, a bootstraps procedure is performed *B* times. The methods sgPLS and joint-sgPLS are then implemented on each bootstrap. The selection rate for variables (resp. group of variables) over the bootstraps are calculated. Rates are considered depending on whether or not the variable is selected by the model computed on true data. Selected variables (resp. group of variable) whose rate is higher than any non-selected variables are kept in the final selection.

#### *Remark 3*

The proposed joint penalization is biconvex but not convex, and thus multiple local minima may exist. The method can then be sensible to the starting point of its algorithm. Some development using several starting points can enhance the probability of reaching a global optimum and some can even ensure it. In dimensionality reduction methods a semidefinite relaxation has been proposed which ensures the convergence [46] at the cost of computational efficiency. Methods relying on random initialization have increased the chances of finding the global optimum but with lower theoretical guarantees. Inheriting such developments for the joint-sgPLS would be interesting for future developments.

#### *Remark 4*

Group sparse dimensionality reduction methods such as sgPLS and joint-sgPLS need to be extended in case of overlapping groups of variables [47]. In the scope of this article, groups of variables are supposed to be disjointed.

### Benchmark methods

Both ASSET and metaSKAT are considered as benchmark methods.

ASSET is a method suited for meta-analysis providing a p-value across studies [6]. The input of the method are single variables summary statistics which are combined by the method. ASSET exhaustively explores subsets of studies for the presence of true association signals that are in either the same direction or possibly opposite directions.

For a given variable $i \in \{1, \cdots ,p\}$ and a given set of studies $m \in \{1, \cdots ,M\}$ the estimate parameters $\{ \beta _{i,m},s_{i,m} \}$ of a linear model on data $X_{\mathbb {M}_m,\cdot }$ and $Y_{\mathbb {M}_m,\cdot }$ and the corresponding statistic $Z_{i,m} = \frac{\beta _{i,m}}{s_{i,m} }$ are computed. Then for each possible subset $S \subset \{1, \cdots ,M\}$, the mean statistic $Z_i(S) = \sum _{l \in S} \sqrt{\pi _l(S)} Z_{i,l}$ is evaluated with $\pi _l(S) = \frac{n_l}{\sum _{l \in S} n_l}$. ASSET seeks for the optimal subset of observations following the criteria $\underset{S \in \mathbb {S}}{\max } |Z(S)|$. A p-value is computed from this final statistic. ASSET relies on statistics at variable level and hence do not propose gene- or pathway-level information. Further, the current version of ASSET provides pleiotropy result for each variant which should be corrected using a FDR correction in order to control possible false positive pleiotropy effect.

SKAT is a method to detect association between rare variants in a region and a phenotype (continuous or binary). It is a supervised test for joint effects of multiple variants in a region on a phenotype. The metaSKAT method can do the same but aggregating several studies. This method outputs a p-value corresponding to a set of variables, for instance a gene or a pathway. The method is based on a weighted sum of SKAT statistics of the different studies [7].

The statistics $S_{m,k} = X_{\mathbb {M}_m,\mathbb {P}_k}^T \tilde{Y}_{\mathbb {M}_m,\cdot }$ is computed where $\tilde{Y}$ of a generalized linear model performed on Y with respect to covariates. Then a weighted sum is computed on these statistics summing among the studies and then following the variables: $Q = \sum _{m=1}^{M} \sum _{k=1}^{K} (w_{m,k} S_{m,k})^2$ where $w_{\cdot ,\cdot }$ are weights that must be chosen. Next, a p-value is computed. The method relies on the square of the statistic and then can detect opposite effects from one study to another.

Unlike metaSKAT, sgPLS and joint-sgPLS ASSET gives one result per variables, and does not give information for a whole group of variables. We can note that both ASSET and metaSKAT are p-value oriented method which allow them to select variables. However, they cannot propose predictions whereas joint-sgPLS can.

### Simulated data

Presented methods are illustrated on simulated data presenting the architecture given in Fig. 1. From one side, SNP genotypes are coded as minor allele counting $\{ 0, 1, 2 \}$ and a certain correlation is expected within a group of SNP from the same linkage disequilibrium block. From the other side, phenotype data are binary and have a true effect from one or more genetic markers. In order to simulate the correlation between SNPs, for a group of variables $\mathbb {P}_k$, a multivariate normal distribution with *n* observations $\varvec{x}^{(continuous)}_k \thicksim \mathcal {N}_{p_k}(\mu _k,\Sigma _k)$ is simulated where $\mu _k$ is a null vector of size $p_k$ and $\Sigma _k$ is a $p_k \times p_k$ matrix with 1 on the diagonal and $\rho _k$, coefficients controlling the correlation between SNPs within a group, outside of the diagonal. A simulation of this variable gives a matrix which represents simulated observations for group of variables *k*. Those blocks are concatenated in a $n \times p$ matrix, $X^{(continuous)}$ that represents the whole data.

In order to have $\{ 0, 1, 2 \}$ genotype data, a discretization is performed. For a given variable $j \in \mathbb {P}_k$, we aim at simulating a SNP variable with a Minor Allele Frequency (MAF), which we note $\text{ MAF}_j$. This MAF means that:

$$\begin{aligned} P(x_j = 0)&= (1- \text{ MAF}_j)^2 \\ P(x_j = 1)&= 2 \text{ MAF}_j (1- \text{ MAF}_j) \\ P(x_j = 2)&= \text{ MAF}_j^2. \end{aligned}$$

To this aim, for a given $\text{ MAF}_j$, quantiles $q_1^{(j)}$ and $q_2^{(j)}$ are chosen such as $P(x_j \le q_1) = (1-MAF_j)^2$ and $P(x_j \le q_2) = (1-MAF_j)^2 + 2 MAF_j(1-MAF_j)$

A discrete genotype, $X^{(discrete)}$, is computed such that

$$\begin{aligned} \begin{aligned} X_{i,j}^{(discrete)} = \left\{ \begin{array}{ll} 0 &{} \text{ if } X^{(continuous)}_{i,j} \le q_1^{(j)} \\ 1 &{} \text{ if } q_1^{(j)} \le X^{(continuous)}_{i,j} \le q_2^{(j)} \\ 2 &{} \text{ if } X^{(continuous)}_{i,j} > q_2^{(j)}, \end{array} \right. \end{aligned} \end{aligned}$$

where $i \in \{ 1 ,\cdots , n \}$ are simulated observations and $j \in \mathbb {P}_k$ is a variable of *k*-th group of variables.

For each observation *i*, a binary phenotype $y_i$ is simulated with a logit model

$$\begin{aligned} \begin{aligned} \text{ logit }(\pi _i) = \text{ log }(\frac{\pi _i}{1-\pi _i}) = \alpha + \sum _{j = 1}^{p} X^{(discrete)}_{i,j} \beta _j, \end{aligned} \end{aligned}$$

where $\pi _i=P(y_i=1 |\text{ data})$, $\beta _j$ for $j \in \{1, \cdots , p \}$ is a regression parameter.

Then different simulations of the process can be performed successively in order to simulate several studies.

## Results

The code used for running the methods is available on github (https://github.com/camilobroc/BMC_joint_sgPLS).

### Simulation

**Table 1:** Values used for the 8 cases of simulated data

Case	Opposite direction effect	Percent of SNPs having an effect in groups having an effect	Total number of observations
1	No	100%	200
2	No	100%	400
3	No	50%	200
4	No	50%	400
5	Yes	100%	200
6	Yes	100%	400
7	Yes	50%	200
8	Yes	50%	400

In this article, simulated genotype has 25 groups of 20 variables. There are then 500 variables and data is composed of two studies with equal number of observations. Combinations of parameters are considered to study a variation of (i) the existence of opposite effects from one observation set to another (ii) the portion of SNPs of the groups having effects (iii) the sample size. Values choice are given in Table 1. Variation (i) permits to see the ability of method at detecting a signal even when opposite effects occur. Variation (ii) allows to observe the influence of intra-group sparsity on the performances of the methods. Variation (iii) shows cases where the signal is easier or harder to retrieve due to the different sample sizes.

The intra-group correlation parameters $\rho _k$ are equal to 0.5 and the MAF is equal to 0.3 for each variable. The first 5 groups have an effect in the model of the simulations. For each group, half of the non-null regression parameters are positives (taken at random) while the other half is negative. In cases where all SNPs have effects (cases 1, 2, 5 and 6), the absolute value of those parameters is set to $\text{ exp }(0.1)$ whereas in cases with half of SNPs having effects (cases 3, 4, 7 and 8), the absolute value of those parameters is set to $\text{ exp }(0.5)$.

**Fig. 2:**
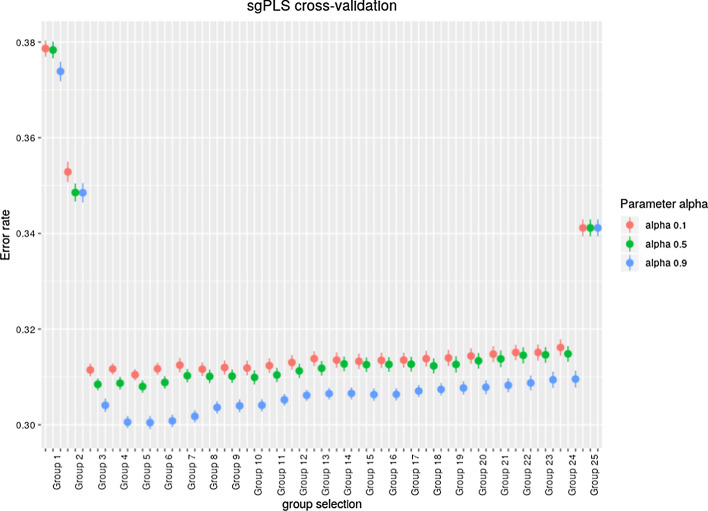
Mean and variance of the error of prediction in cross-validation of sgPLS, for one simulation of case 1 of the simulations. The cross-validation is performed for $\alpha \in \{0.1,0.5,0.9\}$ and for levels of group selection corresponding to $\{1,\cdots ,25\}$

**Fig. 3:**
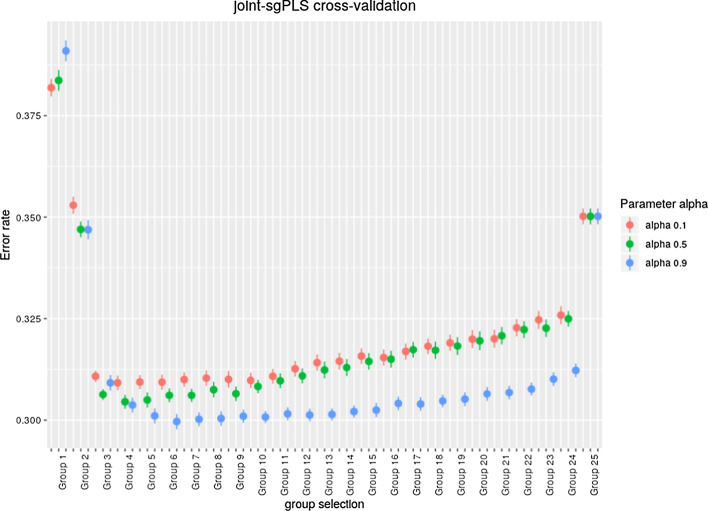
Mean and variance of the error of prediction in cross-validation of joint-sgPLS, for one simulation of case 1 of the simulations. The cross-validation is performed for $\alpha \in \{0.1,0.5,0.9\}$ and for levels of group selection corresponding to $\{1,\cdots ,25\}$

For all methods 50 replications of the data are performed. For the implementation of the sgPLS and joint-sgPLS, penalisation parameters must be chosen similarly to [35]. The penalization parameter $\lambda$ and $\alpha$ are optimized through a *K*-fold penalization procedure with an error of prediction as criteria. Choosing a parameter $\lambda$ is equivalent to set a number of selected groups [35]. In this simulation the grid of number of selected groups $\{ 1,\cdots , 25 \}$ is used and the grid for $\alpha$ is $\{ 0.1 , 0.5, 0.9 \}$. Figures 2 and 3 show the error of prediction performances through a cross-validation procedure of the sgPLS and joint-sgPLS in a simulation of case 1, for different levels for $\alpha$ and different levels of group selection. The observed mean and the variance of the error rate over 50 replications are presented. In the framework of the method the set of parameters corresponding to the lowest error of prediction rate is kept for the model.

For ASSET, sgPLS and joint-sgPLS, the variables selected by the models are compared to the variable having an effect on the true model. For metaSKAT, sgPLS and joint-sgPLS, the group of variables selected by the models are compared to the group of variables having an effect on the true model.

Results of the simulations are presented in Table 1 for sgPLS, joint-sgPLS, ASSET and metaSKAT. The measures of performance are the True Positives (TP), False positives (FP), False Negatives (FN) and True Negatives (TN) (Table 2).

**Table 2:** Performances in terms of mean number of TP, FP, FN and TN over simulation cases 1 to 8 for methods sgPLS, joint-sgPLS, ASSET and metaSKAT

Variable level performances					Group level performances				
	TP	FP	FN	TN		TP	FP	FN	TN
*Simulation case 1*									
sgPLS	46.78	29.24	53.22	370.76	sgPLS	3.54	3.82	1.46	16.18
joint-sgPLS	40.84	27.16	59.16	372.84	joint-sgPLS	3.44	3.78	1.56	16.22
ASSET	29.98	22.14	70.02	377.86	metaSKAT	2.22	1.08	2.78	18.92
*Simulation case 2*									
sgPLS	75.76	139.34	24.24	260.66	sgPLS	4.74	11.9	0.26	8.1
joint-sgPLS	66.12	76.44	33.88	323.56	joint-sgPLS	4.48	7.62	0.52	12.38
ASSET	47.74	25.4	52.26	374.6	metaSKAT	3.14	1.26	1.86	18.74
*Simulation case 3*									
sgPLS	36.58	63.56	13.42	386.44	sgPLS	4.78	4.02	0.22	15.98
joint-sgPLS	31.3	48.04	18.7	401.96	joint-sgPLS	4.5	3.06	0.5	16.94
ASSET	29.46	46.88	20.54	403.12	metaSKAT	3.62	0.96	1.38	19.04
*Simulation case 4*									
sgPLS	42.68	148.78	7.32	301.22	sgPLS	4.88	11.1	0.12	8.9
joint-sgPLS	40.42	115.96	9.58	334.04	joint-sgPLS	4.92	8.54	0.08	11.46
ASSET	35.26	54.68	14.74	395.32	metaSKAT	4.18	1.02	0.82	18.98
*Simulation case 5*									
sgPLS	17.96	36.28	82.04	363.72	sgPLS	1.46	4.32	3.54	15.68
joint-sgPLS	43.44	25.2	56.56	374.8	joint-sgPLS	3.46	3.5	1.54	16.5
ASSET	30.58	22.48	69.42	377.52	metaSKAT	2.2	0.98	2.8	19.02
*Simulation 6*									
sgPLS	75.76	139.34	24.24	260.66	sgPLS	4.74	11.9	0.26	8.1
joint-sgPLS	66.12	76.44	33.88	323.56	joint-sgPLS	4.48	7.62	0.52	12.38
ASSET	47.74	25.4	52.26	374.6	metaSKAT	3.14	1.26	1.86	18.74
*Simulation case 7*									
sgPLS	13.2	74.96	36.8	375.04	sgPLS	2.04	6.5	2.96	13.5
joint-sgPLS	35.62	94.24	14.38	355.76	joint-sgPLS	4.58	7.02	0.42	12.98
ASSET	29.18	45.5	20.82	404.5	metaSKAT	3.54	0.92	1.46	19.08
*Simulation case 8*									
sgPLS	14.3	72.28	35.7	377.72	sgPLS	1.88	5.76	3.12	14.24
joint-sgPLS	39.12	99.06	10.88	350.94	joint-sgPLS	4.9	7.18	0.1	12.82
ASSET	34.04	56.14	15.96	393.86	metaSKAT	4.22	0.86	0.78	19.14

Considering cases 1 and 2, we can see that ASSET and metaSKAT have FP lower than TP in opposition to sgPLS and joint-sgPLS. They are then more conservative than the two later methods. We can see that overall, each model performs better when the number of observations is higher (200 against 400). We can see that when the intrasparisity is set to 50 % (cases 3, 4, 7 and 8) rather than 100 % (cases 1, 2, 5 and 6), variable-level results for ASSET sgPLS and joint-sgPLS are inflated by more than a half. This may be due to the fact that the methods struggle to differentiate the effect of variables within a same group. The gene-level results are similar whichever the intrasparsity is for metaSKAT, sgPLS and joint-sgPLS. Cases 1, 2, 3 and 4 have effect in the same direction among studies while cases 5,6,7 and 8 show effects in opposite directions. We can see that when effects are in the same direction or opposite direction, sgPLS can compete with joint-sgPLS and with other benchmark methods while being the least conservative. On the other hand, when effects are in different directions, sgPLS performances fumble whereas other methods keep a similar TP/FP ratio. Comparing closely ASSET to joint-sgPLS, we can see that joint-sgPLS have always a higher TP and the largest difference can be seen when all variables are involved within a group (cases 1, 2, 5, 6). This is probably due to the fact that joint-sgPLS can draw information at the group-level to infer single variable results. Comparing closely metaSKAT to joint-sgPLS, we can see that both methods can retrieve a large amount of groups participating to the effect. The method joint-sgPLS have always a higher TP in each cases. In cases 1 and 5, metaSKAT TP is especially low. Those are cases with the smallest number of observations and with small regression parameters $\beta _j$ and hence where the intensity of the signal is the lowest.

Overall, we can see that sgPLS and joint-sgPLS have competitive performances for detecting effect in the same direction while joint-sgPLS is the method with the best performance for detecting opposite effects. Furthermore sgPLS and presented joint-sgPLS have the merit of giving single variable results and group results in the same model. This allow variable-level results to be enhanced by the group a priori information.

### Pleiotropy investigation on breast and thyroid cancer

The developed statistical approaches were applied to real data in order to enrich our insights about the genetic mechanisms involved in carcinogenesis of thyroid and breast cancers. Thyroid and breast cancers share some similarities in their biology: both are more frequent in women, are influenced by hormonal and reproductive factors and are hormonally-mediated. Moreover, individuals diagnosed with breast cancer are more likely to develop thyroid cancer as a secondary malignancy than patient diagnosed with other cancer types, and vice-versa [48]. Genetic factor contributing to the incidence of breast cancer have been extensively studied, and it is known that genetic variants explain approximately 49 percent of the familial risk to develop this disease. Using GWAS, 313 risk variants were identified for breast cancer [49]. On the other hand, GWAS studies on thyroid cancer have been scarce, due to the lesser incidence of this disease as well as the lack of data. However, it has been shown that thyroid cancer is the only cancer for which genetic factors contribute more than environmental factors [50]. Only 4 loci have been associated with thyroid cancer risk and have been replicated in other studies [51]. One of them, 2q35, was also previously reported to increase risk of breast cancer [52]. To date, no study has been conducted to identify common genetic factors between breast and thyroid cancer. Exploring the genetic relationship between the two cancers would help to elucidate the common mechanisms between both disease and could permit to improve their diagnostic and therapeutic management.

We propose to illustrate the methods on real datasets, by investigating the pleiotropic effect of genetic variants from candidate pathways in breast and thyroid cancers.

Beluhca dataset includes data from CECILE, a french case-control study on breast cancer (1 125 cases, 1 172 controls) and from CATHY a french case-control study on thyroid cancer (463 female cases and 482 female controls). All these individuals were genotyped using a customized microarray including 8 716 genetic variants from 28 candidate pathways (648 genes) selected from KEGG database and from a literature review (SNPs are located at +/− 50 kb from the gene boundaries). After quality controls, we retained 6 677 SNPs available for both type of cancers. Missing values were imputed using the median among cases or controls and data were centered to $\mu = 0$. When 2 SNPs were correlated at $r^2=1$, one of the SNP was removed and couple of extremely correlated ($r^2 > 0.98$) SNPs belonging to same genes were eliminated.

As group sparse dimensionality reduction methods such as sgPLS and joint-sgPLS need to be extended in case of overlapping groups of variables [47], 10 non-overlapping pathways were selected and only the 3766 SNPs related to those groups were kept in the final database. After all these preprocessing, the new dataset is composed of 3766 SNPs, grouped in 337 non-overlapping genes and 10 non-overlapping pathways. The list of the pathways and genes is displayed in Tables 5 and 6 in Appendix 1.

**Fig. 4:**
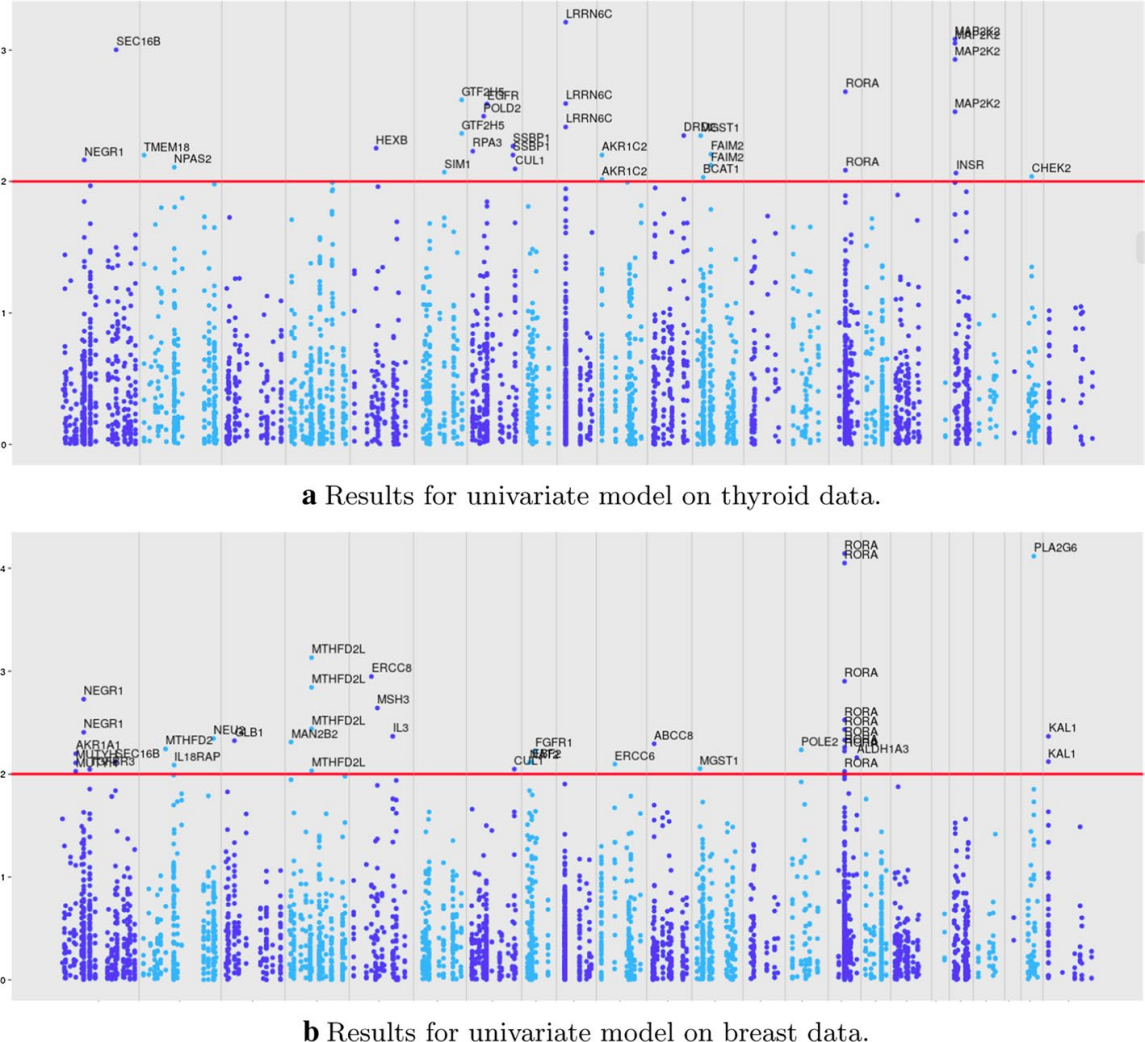
Score for association of SNPs with the outcome for univariate model. The score is computed as $- \text{ log}_{10} (p)$ where *p* is the p-value. The red line corresponds to the threshold 0.01. The alternation of blue colors shows the different chromosomes

The methods implemented in this article are: ASSET, metaSKAT, sgPLS and joint-sgPLS. For metaSKAT, sgPLS and joint-PLS, SNP-level, gene-level and pathway-level results are given by the methods whereas ASSET gives only SNP-level results. Hence, in the case of ASSET, genes corresponding to selected SNPs are considered. For each SNP *i*, an univariate logistic model for gene-disease association can be considered separately for thyroid data and breast data (thyroid and breast cancer, Fig. 4).

**Fig. 5:**
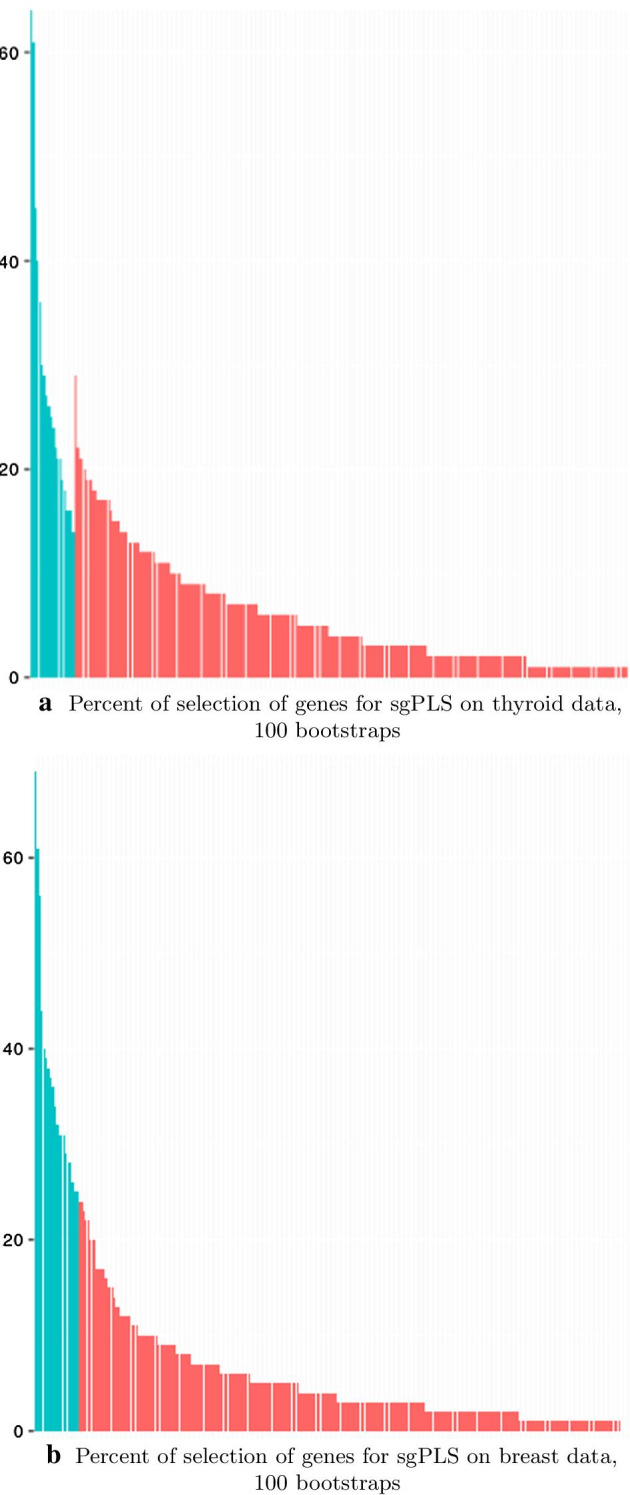

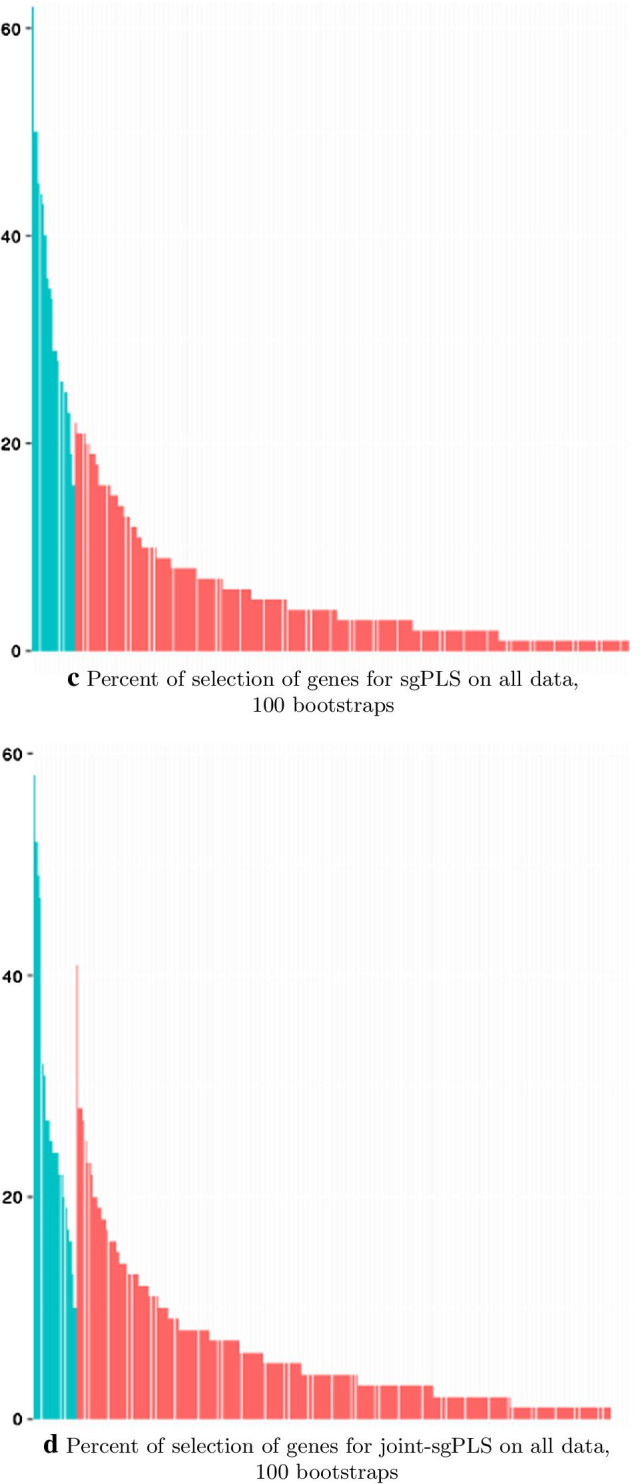
Percent of selection of genes for sgPLS and joints sgPLS on 100 bootstraps. **a** sgPLS on thyroid data. **b** sgPLS on breast data. **c** sgPLS on both data. **d** joint-sgPLS. Genes selected on original data (preselected ones) are in blue while other genes (non-preselected ones) are in red

**Fig. 6:**
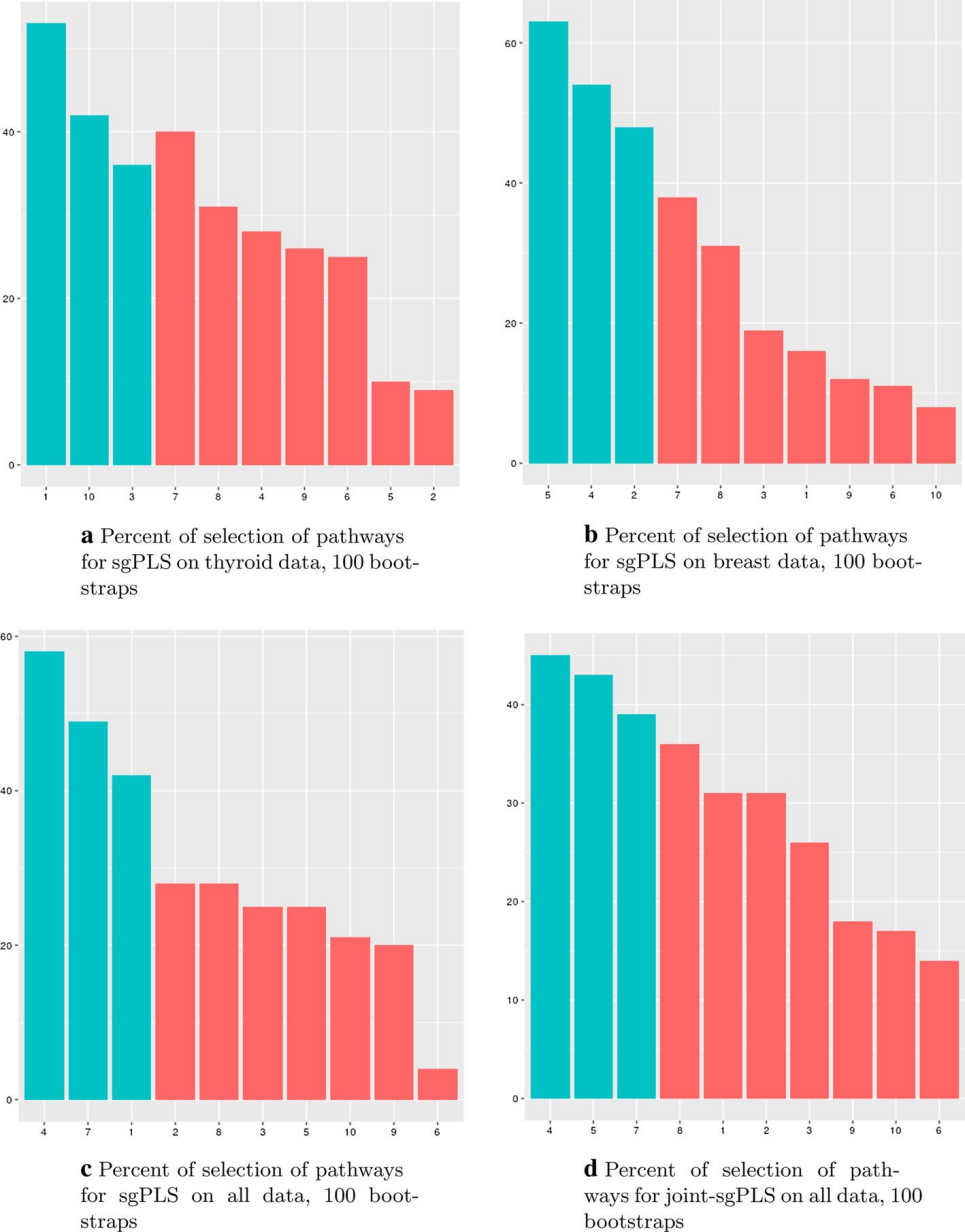
Percent of selection of pathways for sgPLS and joints sgPLS on 100 bootstraps. **a** sgPLS on thyroid data. **b** sgPLS on breast data. **c** sgPLS on both data. **d** joint-sgPLS. Pathways selected on original data (preselected ones) are in blue while other pathways (non-preselected ones) are in red. The pathways are noted: (1) Cell cycle (2) Circadian rhythm (3) Folate metabolism (4) Other glycan degradation (5) Obesity and obesity-related phenotypes (6) DNA repair (7) Metabolism of xenobiotics (9) Precocious or delayed puberty (10) Inflammatory response

As it has been presented before, for sgPLS and joint-sgPLS, a calibration of the parameters is generally performed through a cross-validation procedure. This process relies on the definition of a measure of performance: the error of prediction of the model. However, in genetic studies, the effects are small and the prediction performances based on genetic units are usually very low. The prediction performance of sgPLS and joint-sgPLS are not different enough from one set of penalization parameters to another. In order to facilitate the interpretation, we present the results for calibration parameters set to 20 genes and 3 pathways and $\alpha =0.5$. We explore the stability of the methods using the bootstrap strategy described in the section method. Figures 5 and 6 present this rate for preselected and non-preselected features. A gene and resp. a pathway is kept in the final selection if and only if it is preselected and its rate of selection among the bootstraps is higher than any other gene (resp. pathway) that is not preselected. We can see that for joint-sgPLS less genes are selected than for other methods (4 against resp. 20 and 18 for metaSKAT and sgPLS on both data).

Results of the selection are presented in Table 3 where the name of genes and pathways is presented. “sgPLS single” stand for the use of the sgPLS on thyroid and breast data separately while “sgPLS both” stands for the use of the method on a concatenation of both data set standardizing by study. Only genes that are selected by at least one method are presented. No genes from metabolism of xenobiotics pathway have been selected through all methods. We can see that methods focusing on SNP-level information select gene from one of the study but never both studies at the same time except for *INSR* which is selected for both studies for SKAT. This genes is not selected by meta-analysis methods. Genes selected by group-level methods (ASSET, metaSKAT, sgPLS, joint-sgPLS)) that are not selected by variable-level methods are: PTEN, RORA, MSH3, IL18RAP,GNPDA2, LRRN6C, NEGR1, NR3C1, SEC16B, HEXA, HEXB, MAN2B2, NEU2, TGBR3, NMNAT2, CYP2C18, CYP2C19, MGST1. Those genes are good candidates for further investigations as they are not selected by study by study analyses but are selected by meta analyses. We can note that 5 out the 8 genes selected for Obesity and obesity-related phenotypes pathway and all genes selected for Other glycan degradation are part of those genes. Those pathways would not have been as much hightlighted without meta-analyses. Genes selected for thyroid data sets and selected by meta-analyses are: MAP2K2, GTF2H1 and CYP2F1. Those genes are then related to thyroid cancer but meta-analyses suggest they may be involved with breast cancer in a common effect. Genes selected for breast data and selected by meta-analyses are: PLAG2G6, ERCC3, ERCC6, MUTYH, MTHFD2, IL13, NAT2. Meta-analyses suggest that these genes may also be involved with thyroid cancer in a common effect. We can see that joint-sgPLS selects a lower number of genes (resp.4) compared to ASSET, metaSKAT and sgPLS (resp. 19, 20, 18). Method sgPLS and joint-sgPLS select the glycan pathway and folate metabolism pathway and sgPLS selects also cell cycle pathway. PLS methods suggest that pathway-level effect could be involved.

#### *Remark 5*

Results based on different choice of calibration parameters for sgPLS and joint-sgPLS (50, 100 genes and 5 pathways) showed similar patterns.

**Table 3:**
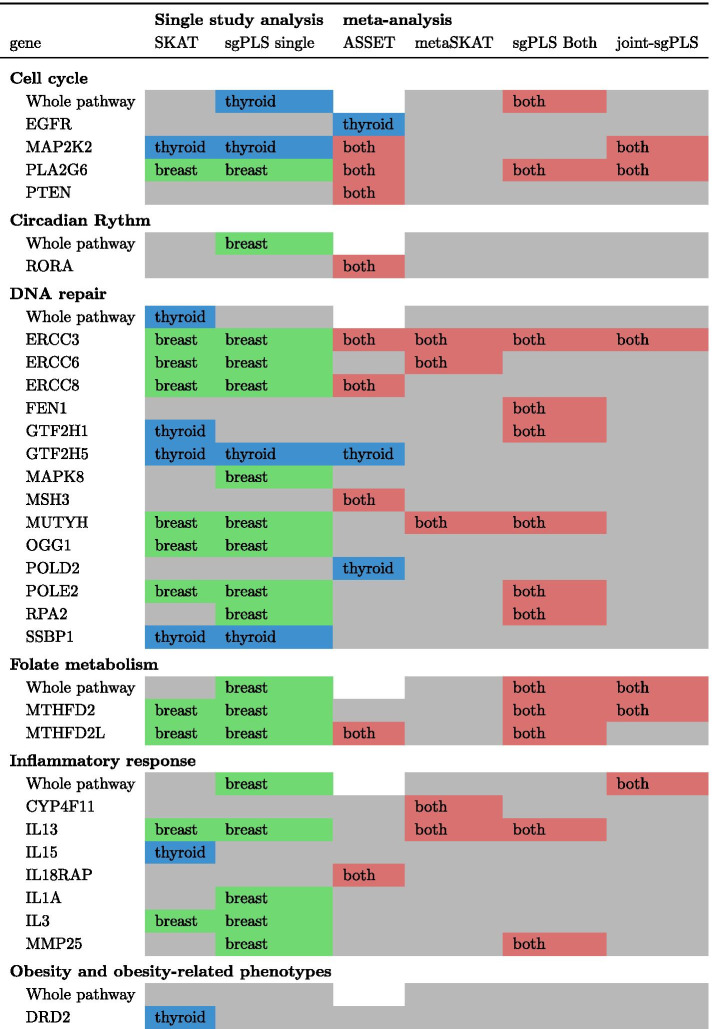

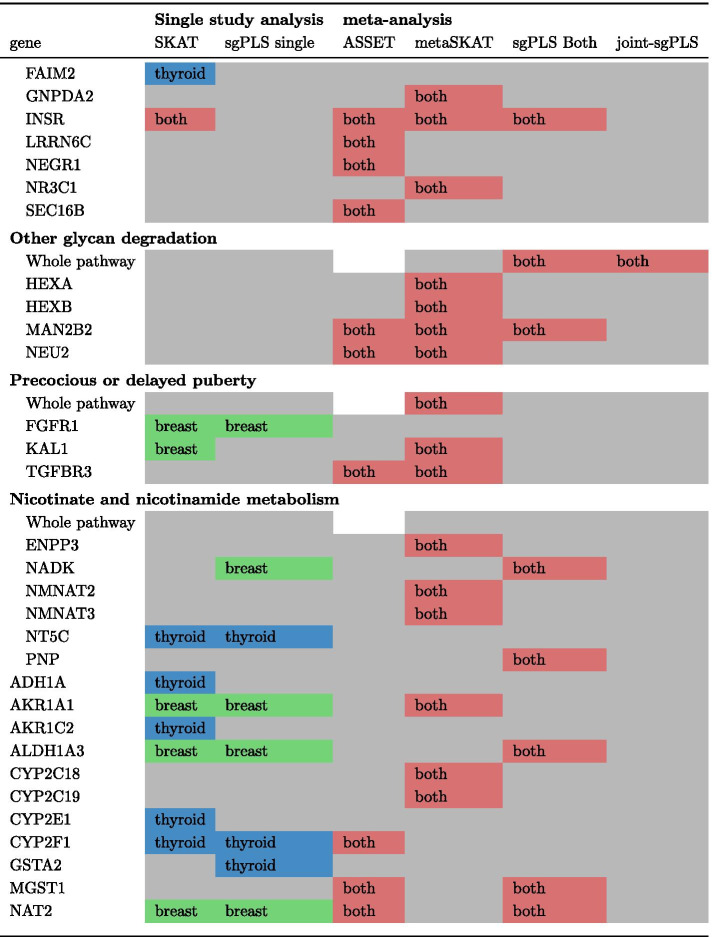
Selected data sets in terms of genes and pathways. Selection of resp. thyroid data set, breast data sets and both data set is represented in resp. blue, green and read

### Computational performances

Computation performances are presented on simulation cases 1 and 2 which represent data having 500 predictors and 1 output (Table 4). The number of observations is respectively $n = 200$ and $n = 400$. The methods sgPLS and joint-PLS penalization hyperparameters are estimated with the grid used for the simulation. Mean running times over 50 replications are given.

**Table 4:** Computational performances in seconds of ASSET, metaSKAT, sgPLS and joint-sgPLS for case 1 (n=200) and case 2 (n=400)

n	200	400
ASSET	1.21	1.69
metaSKAT	0.22	0.59
sgPLS	18.29	37.03
joint-sgPLS	31.27	67.80

We can see overall that has the smallest running time. The methods sgPLS and joint-sgPLS have the most expensive computational. This is due to the estimation of the penalization parameters as hyperparameters. However, this calculus consist in successive applications of the same method. It can then be paralellized.

## Discussion

In this article, the properties of the joint-sgPLS are presented and are compared to the classical sgPLS, the ASSET method and metaSKAT. The methods ASSET, metaSKAT and joint-sgPLS are suited for meta-analyses whereas sgPLS is not. ASSET only gives variable-level results whereas metaSKAT and joint-sgPLS can assess group-level results. However, joint-sgPLS is the only method proposing to link in a same model variable results and group results. The method have then more interpretability while have competitive or superior performances over simulations compared to benchmark methods. Hence, joint-sgPLS seems perfectly suited for meta-analysis where effects in opposite directions can exist which invite us to pursue further investigation with it in complex studies for genetic epidemiology such as pleiotropy.

## Conclusion

We do believe that further investigation can be done on the same subject. In this article, sgPLS and joint-sgPLS have been applied with one component, but several components could be considered. This could lead to the selection of variables that are orthogonal to the selection of the first component but that have still a large participation to the covariance matrix.

We acknowledge that on the application the stability of the method is an important point due to the fact that the cross-validation procedure is not satisfying for choosing the parameters of penalization. One improvement could consist in exploiting different the criteria of the procedure (the error prediction) with, for instance, stability measures [53]. Another improvement could consist in adaptating the adaptative Lasso [54] for our method which could bypass the stability questions.

Presented method uses a group architecture, but adding group-sub-group architecture is an interesting path of investigation for taking into account gene- and pathway-level information at the same time. The methods sgsPLS ( [36]) already offers a sparse partial least squares framework with group and subgroup architecture which is an extension of the sgPLS. A similar work could lead to a promising joint-sgsPLS.

In order to advance on the application, this study should be replicated on a larger data base. Particularly, thyroid cancer has been less studied than breast cancer, and data for thyroid are still scarce in this application. Other cases of pleiotropy could be investigated, for instance for the case where the phenotype is multivariate for each subject. The joint-sgPLS is suitable for any kind of phenotype, continuous or qualitative. R code is available from the author to reproduce the results and is available on github (https://github.com/camilobroc/BMC_joint_sgPLS).

## Data Availability

Code generating simulated data which has been analysed in this article is available at https://github.com/camilobroc/BMC_joint_sgPLS. It is designed for R software (version 3.6.3 and higher). The application dataset “Beluhca” that has been used during the current study is available from the authors on reasonable request.

## Appendix 1: Tables of genes and pathways

See Tables 5, 6.

**Table 5:** First pathways and their corresponding genes.

Gene	Count
(1)	
ENPP1	27
NMNAT3	25
AOX1	24
NMNAT2	21
ENPP3	15
BST1	14
CD38	11
NT5M	10
NT5C1A	9
NT5C2	8
PNP	8
NAMPT	7
NNMT	7
NT5C3	7
NADSYN1	6
NNT	6
NT5E	5
NMNAT1	4
NUDT12	4
QPRT	4
NT5C1B	3
NADK	2
NT5C	2
(2)	
RORA	282
NPAS2	60
RORB	34
ARNTL	23
CUL1	22
BTRC	13
RORC	13
PER3	12
CLOCK	11
PER2	11
CRY2	9
CSNK1E	9
BHLHE40	8
FBXW11	8
CRY1	7
FBXL3	7
NR1D1	7
TIMELESS	7
PER1	5
SKP1	4
BHLHE41	3
CSNK1D	3
RBX1	2
(3)	
MTHFS	19
MTHFD1	11
MTHFR	11
MTHFD2L	9
MTHFD2	1
(4)	
GLB1	22
MAN2B2	18
ENGASE	13
HEXB	12
MANBA	9
AGA	8
FUCA2	8
NEU2	5
FUCA1	3
HEXA	3
NEU3	3
GBA	2
MAN2B1	2
NEU1	2
NEU4	1
(5)	
LRRN6C	197
FTO	122
NEGR1	93
SCARB1	33
ABCC8	31
SEC16B	25
LEPR	23
MAP2K5	23
NR3C1	18
FAIM2	17
DRD2	16
PPARG	16
SIM1	14
FANCL	12
GHRL	12
ADIPOQ	11
CRHR2	11
GNB3	10
INSR	10
GPRC5B	9
MC3R	9
PCSK1	9
TFAP2B	9
TNF	9
UCP1	9
(5)	
CRHR1	8
ETV5	8
IL1RN	8
LDLR	8
HTR2C	7
MCHR1	7
TMEM18	7
BDNF	6
KCTD15	6
UCP2	6
ACE	5
ADRB2	4
IL6	4
LEP	4
MC4R	4
PLIN	4
PTPN11	4
UCP3	4
GNPDA2	3
NR0B2	3
RETN	3
CCL5	2
LEPROTL1	2
SH2B1	1
(6)	
RPA3	25
NEIL3	24
XRCC5	21
EXO1	19
MSH3	19
NEIL2	17
RPA1	17
BRCA2	16
RAD23B	16
RFC3	14
PARP4	13
POLD3	12
RFC5	12
CHEK1	11
CHEK2	11
MSH6	11
TERT	10
CASP7	9
MNAT1	9
PARP1	9
RFC1	9
XPC	9
CASP3	8
ERCC5	8
ERCC6	8
(6)	
TDG	8
XRCC1	8
DDB2	7
ERCC8	7
MSH2	7
PMS2	7
POLD1	7
POLE	7
RFC4	7
XRCC2	7
XRCC3	7
CDK2	6
GTF2H1	6
LIG1	6
MUTYH	6
PARP2	6
POLD2	6
POLE2	6
TP53	6
XPA	6
BRCA1	5
CDK7	5
CHRNA4	5
CUL4B	5
ERCC3	5

The number of SNPs for each gene is presented: The pathways are (1) Nicotinate and nicotinamide metabolism (2) Circadian rhythm (3) Folate metabolism (4) Other glycan degradation (5) Obesity and obesity-related phenotypes (6) DNA repair

**Table 6:** Last pathways and their corresponding genes.

Gene	Count
(6)	
GTF2H5	5
LIG3	5
MAPK8	5
NTHL1	5
OGG1	5
RAD50	5
RPA4	5
APEX1	4
CDKN1A	4
ERCC2	4
FEN1	4
GTF2H4	4
MBD4	4
POLE3	4
RAD51	4
RFC2	4
UNG	4
CDKN2D	3
CETN2	3
CUL4A	3
ERCC1	3
MLH1	3
MPG	3
POLB	3
POLE4	3
(6)	
POLL	3
RPA2	3
SSBP1	3
CCNH	2
ERCC4	2
GTF2H3	2
HMGB1	2
(7)	
UGT1A8	58
UGT2A1	29
MGST2	22
CYP2C8	17
AKR1C2	16
CYP2C9	15
CYP2B6	14
EPHX1	14
MGST3	14
AKR1C4	13
COMT	12
CYP2C19	12
CYP2S1	12
CYP2C18	11
ADH1B	10
ADH7	10
GSTA4	10
GSTZ1	10
MGST1	10
NAT2	10
ADH1C	9
AHR	9
NAT1	9
ADH6	8
AKR1C3	8
(7)	
CYP1B1	8
UGT2B4	8
ADH4	7
ADH5	7
AKR1C1	7
ALDH3B1	7
CYP2E1	7
NQO1	7
ALDH1A3	6
CYP2F1	6
DHDH	6
SOD2	6
GSTA2	5
GSTM3	5
GSTO2	5
GSTP1	5
CYP1A1	4
CYP1A2	4
CYP3A43	4
GSTA3	4
GSTM4	4
UGT2B7	4
AKR1A1	3
ALDH3B2	3
CYP2A6	3
(7)	
CYP3A4	3
CYP3A7	3
GSTM2	3
GSTM5	3
UGT2A3	3
UGT2B11	3
ADH1A	2
CYP2D6	2
(8)	
TGFBR3	77
EBF2	60
BCAT1	51
VDR	34
KAL1	26
TM7SF3	19
CASC1	17
FGFR1OP2	12
FGFR1	11
KRAS2	10
CCR3	8
KISS1	8
PROK2	7
LIF	5
PROKR2	5
PTH1R	4
NKX2-1	2
(9)	
TGFB2	22
IL18RAP	13
CYP4F11	12
EPHX2	12
IL7	11
IGFBP1	9
IGFBP3	9
IL17A	9
IL10	8
IGFBP4	7
IL15	7
MMP25	7
IL16	6
IL12A	5
IL13	5
IL18	5
IL19	5
IL2	5
IL9	5
PLA2G4B	5
IL3	4
TGFB1	4
IL1B	3
IL4	3
IL1A	2
IL23A	1
10)	
EGFR	103
CCND3	29
MAPT	19
MAP2K4	15
EGF	11
MAP2K2	11
GSK3B	8
PLA2G6	8
PTEN	8
MAP2K1	6
MYBL2	6
AKT1	5
MAP2K3	5
FGFR3	3
MAP2K7	3
MAPK12	3
TP53I3	3
CCNA2	2
MAPK7	2

The number of SNPs for each gene is presented. The pathways are (6) DNA repair (7) Metabolism of xenobiotics (8) Precocious or delayed puberty (9) Inflammatory response (10) Cell cycle

## Abbreviations

GWAS: Genome-wide association study
SNP: Single nucleotide polymorphism
MAF: Minor allele frequency
PLS: Partial least squares
sPLS: sparse partial least squares
sgPLS: sparse group partial least squares
sgsPLS: sparse group sparse partial least squares
SKAT: SNP Kernel Association Test
TP: True positive
FP: False positive
FN: False negative
TN: True negative
